# Avoiding restorative proctocolectomy for colorectal cancer in patients with ulcerative colitis based on preoperative diagnosis involving p53 immunostaining: report of a case

**DOI:** 10.1186/s12957-015-0540-7

**Published:** 2015-03-26

**Authors:** Haruki Sada, Manabu Shimomura, Takao Hinoi, Hiroyuki Egi, Koji Kawaguchi, Takuya Yano, Hiroaki Niitsu, Yasufumi Saitou, Hiroyuki Sawada, Masashi Miguchi, Tomohiro Adachi, Hideki Ohdan

**Affiliations:** Department of Gastroenterological and Transplant Surgery, Applied Life Sciences, Institute of Biomedical and Health Science, Hiroshima University, 1-2-3 Kasumi, Minami-ku, Hiroshima 734-8551 Japan

**Keywords:** Sporadic cancer, Ulcerative colitis, p53 overexpression

## Abstract

The standard operation for colitic cancer in ulcerative colitis (UC) is restorative proctocolectomy; however, sporadic colorectal cancer (CRC) can coincidentally arise in patients with UC and the optimal procedure remains controversial. Therefore, it is crucial to preoperatively determine whether the CRC in UC is a sporadic or colitic cancer. We report a case of avoiding proctocolectomy for sporadic CRC in a patient with UC based on preoperative diagnosis involving p53 immunostaining. A 73-year-old man with CRC in UC had undergone sigmoid colectomy with lymphadenectomy because of the submucosal deep invasion pathologically after endoscopic mucosal resection. The cancer was diagnosed sporadic cancer preoperatively not only based on the endoscopic, clinical, and histological patterns but also that the colon epithelium was unlikely to develop dysplasia as the circumference and unaffected UC mucosa did not detect p53 protein overexpression. Recent reports have shown that the immunohistochemical detection of p53 protein overexpression can be useful for a differential diagnosis and as a predictor of dysplasia and colitic cancer. The analysis of p53 mutation status based on immunostaining of p53 protein expression in the unaffected UC mucosa can be useful for the decision regarding a surgical procedure for CRC in patients with UC.

## Background

Patients with chronic extensive ulcerative colitis (UC) are at high risk for developing colorectal cancer (CRC) or dysplasia, which is supposed to develop along an inflammation-dysplasia-carcinoma sequence [1]. CRC in UC is a serious complication that can be life-threatening. However, sporadic adenomas or adenocarcinomas can arise coincidentally, especially in elderly patients with UC. Differential diagnosis between colitic cancer and sporadic cancer is an important issue in critical practice, because the treatment method differs considerably between these conditions [2]. Although restorative proctocolectomy is recognized as the standard procedure for colitic cancer, the optimal procedure for sporadic CRC remains questionable and partial resection can be adequate, as the functional quality of life differs substantially between patients with and without proctocolectomy. A recent report has shown that for CRC in UC, restorative proctocolectomy could be avoided because of the diagnosis of sporadic cancer based on endoscopic and histological findings [3]. The differences between colitic and sporadic cancer have been investigated based on endoscopic, clinical, and histological findings [2,4]. Even in such studies, clearly distinguishing sporadic and colitic cancer is difficult. Additionally, the immunohistochemical detection of p53 protein overexpression can be useful for the differential diagnosis and as a predictor of dysplasia and colitic cancer [5-7]. We herein report that, for the first time, colon cancer in UC was resected partially not only based on endoscopic, clinical, and histological findings but also by detecting unaffected UC mucosa not detecting p53 protein overexpression.

## Case presentation

**Figure 1:**
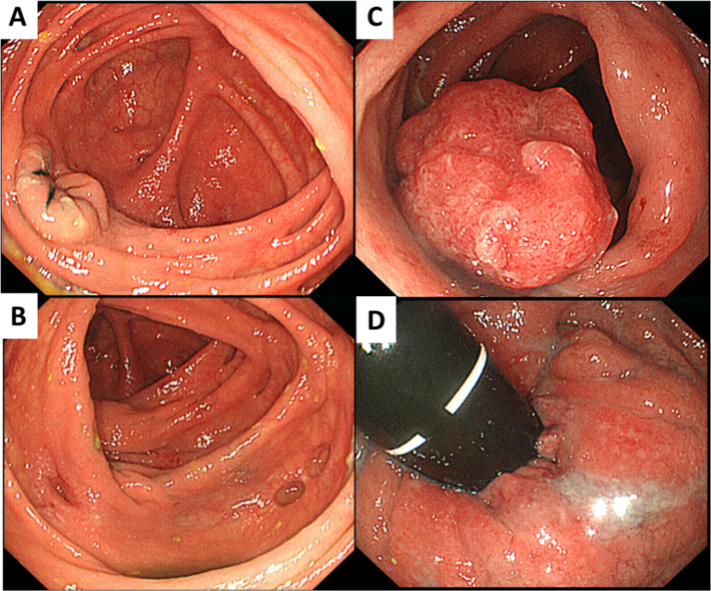
**Initial endoscopic findings.** Colonoscopy showed mild mucosal inflammation throughout the cecum to the left-sided colon **(A,B)** and moderate inflammation from the sigmoid colon to the rectum **(C,D)**. The findings also included a well-circumscribed raised tumor, which was revealed to have a type IV to VI pit pattern, 15 mm in diameter, in the sigmoid colon **(C)**.

**Figure 2:**
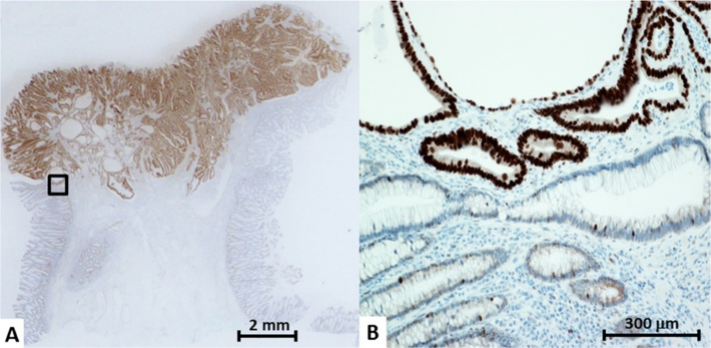
**Histopathological findings for the endoscopic resection on immunohistochemistry for p53 protein. (A)** The cancer lesion shows diffuse positivity on p53 immunostaining. **(B)** The boundary between cancer and circumference mucosa is shown. The circumference mucosa adjoining to cancer does not show any dysplasia. Only a few weak positive cells are exhibited in the non-neoplasia mucosa.

A 73-year-old man was referred to our hospital complaining of having had bloody stools five to seven times/day for 1 month. He had no noteworthy medical or familial history, including inflammatory bowel diseases. Colonoscopy showed moderate mucosal inflammation throughout the rectum to the sigmoid colon and multiple erosions from the sigmoid colon to the cecum. The findings also included a well-circumscribed raised tumor, which was revealed to have a type IV to VI pit pattern, 15 mm in diameter, in the sigmoid colon (Figure 1). Step biopsying was performed at 18 points from the rectum to the terminal ileum, there being no findings of dysplasia lesions microscopically. The pathological findings revealed UC Matts’ grades 3 to 4 in the rectum to the sigmoid colon and Matts’ grades 1 to 2 in the left-sided colon to the cecum. He was given mesalazine (2,400 mg/day) as the initial medical treatment. Endoscopic mucosal resection was also performed to remove the sigmoid colon tumor at 1 month after the initial diagnosis. Histological examination showed a 15 × 10-mm well-differentiated adenocarcinoma with submucosal deep invasion (4,000 μm) that detect diffusely p53 protein accumulation in immunostaining. There was no intravenous or lymphatic invasion microscopically, and the horizontal and vertical margins were negative. The circumference mucosa around the cancer lesion did not show dysplasia, and the circumference and unaffected UC mucosa were considered negative for the p53 protein overexpression because only a few weakly positive cells were detected (Figure 2).

For this case, additional surgical resection with lymphadenectomy was required because of the submucosal deep invasion. At 3 months after the initial diagnosis of UC, the bloody stools had disappeared, and clinical improvement was attained with mesalazine (3,600 mg/day) and prednisolone (15 mg/day). Also, an endoscopic examination showed improvement and Matts’ grade was 2 to 3 in the rectum to the sigmoid colon pathologically. We performed laparoscopy-assisted sigmoid colon resection with lymph node dissection. The decision to perform partial resection was made for two reasons. First, the initial diagnosis was sporadic cancer. Second, his UC was well controlled with no precious history, and his colon was not likely to develop dysplasia or colitic cancer. Sporadic cancer was diagnosed based on the distinction from colitic cancer: late onset and short duration of UC clinically, no characteristic endoscopic findings of colitic cancer, no poorly differentiated adenocarcinoma or signet-ring cell carcinoma, and no detection of dysplasia pathologically. In addition, his UC was well controlled with medical treatment and the unaffected UC mucosa did not detect p53 protein overexpression, which has been reported a predictor of dysplasia and colitic cancer within the next few years. The resected specimen showed no residual carcinoma tissue at EMR sites in the sigmoid colon. The examined 38 lymph nodes showed no evidence of a metastatic carcinoma. The postoperative clinical course was uneventful. We added granulocytapheresis (GCAP) therapy at 1 week after operation, in order to prevent the UC from getting worse and to reduce the amount of steroids. One year after the operation, there was no evidence of cancer recurrence, and the UC was in the remission state with only mesalazine (3,600 mg/day), that is, no steroid therapy.

## Discussion

We have shown the detection of p53 protein overexpression in unaffected UC mucosa to be valuable to the decision regarding a surgical procedure for CRC in patients with UC. The p53 gene is a member of a family of tumor suppressor genes, and inactivation of this protein plays a crucial role in the emergence and further progression of a multitude of human malignancies including carcinomas of the colon and rectum [6]. Most colitis-associated CRC undergoes an inflammation-dysplasia-carcinoma sequence, as opposed to the sporadic CRC, which undergoes an adenoma-carcinoma sequence [4]. There are various differences in the timing and frequency of molecular changes between sporadic and colitic cancer. In colitic cancer, mutations and a loss of heterozygosity of p53 gene are early events in UC mucosa, often occurring before dysplasia is detected. However, in sporadic cancer, p53 mutations are relatively late events in the progression of adenocarcinomas [8,9]. Ullman and Itzkowitz reported that oxidative stress was likely to be involved in these differences. And reactive oxygen and nitrogen species produced by inflammatory cells could affect regulation of genes that encode factors that prevent carcinogenesis, transcription factors, or signaling proteins [10].

Two previous studies investigated the prevalence of a p53 protein overexpression for immunohistochemistry [11,12]. Diffuse pattern, wherein strongly positive cells exist in most areas of the tubules, and nested pattern, wherein moderate to strongly positive cells are aggregated in restricted areas of the tubule, were presumed to reflect mutant forms of p53 protein and were defined as overexpression of p53 protein [11]. Regarding invasive CRC, colitic cancer with UC was expressed in 90.9% and 100%, and sporadic cancer cases without UC in 54.5% and 59.5%. Additionally, in low-grade dysplasia, the prevalences were 12.6% and 71.4%, and in high-grade dysplasia 80% and 100%, compared with 5.6% in sporadic adenomas with and without UC and 0% in non-neoplastic UC mucosa even in the active phase. A fairly wide range of overexpressed p53 prevalence in low-grade dysplasia was observed. Sato *et al*. assessed that these discrepancies might be due to the differences between antibodies and antigen retrieval methods. Moreover, the precise nature of low-grade dysplasia is still controversial and low-grade dysplasia diagnosed histologically can consist of heterogeneous lesions [11]. Thus, p53 immunostaining was considered to be a helpful method for making a precise differential diagnosis of dysplasia, distinguishing it from atypical epithelium due to inflammatory or regenerating changes and from sporadic adenomas, coincidentally complicating UC [11,12].

Also, Lashner *et al*. evaluated colonic biopsy specimens in immunohistochemical staining defining positive for the p53 mutation if more than 5% of the epithelial cells in a biopsy specimen exhibited dark brown intranuclear staining. And they found p53 mutations developed approximately 8 months before low-grade dysplasia, 26 months before high-grade dysplasia, and 38 months before cancer. In addition, patients who tested positive for p53 mutations were more than four times as likely than p53-negative patients to have dysplasia or cancer [5]. Furthermore, Takaku *et al*. have described that if histologically non-neoplastic-appearing mucosa with p53 protein overexpression harbors p53 mutations, this may be an early warning sign for UC patients at risk of developing carcinomas [6]. Thus, the detection of p53 protein overexpression can be one of the valuable complementary tests for surveillance [5].

It is of clinical importance that the p53 gene mutations do not always coincide with p53 protein overexpression. Of all the p53 mutations in human cancer, 80% are missense mutations that result in a prolonged half-life of the mutant p53 protein, allowing their detection by immunohistochemistry. Stop codon or nonsense mutations constitute less than 20% of p53 mutations in human tumors. These mutations cause a shortened, unstable, or absent p53 protein product resulting in a completely negative p53 expression [7,13]. Thus, single-strand conformation polymorphism and PCR currently are the most precise methods for identifying p53 suppressor gene mutations and a loss of heterozygosity; however, these types of analyses are too expensive and time-consuming for routine use in cancer surveillance. In contrast, immunohistochemistry is much less expensive and is technically easy and monoclonal antibodies are readily available [5]. For these reasons, many previous studies investigated p53 mutational status by immunohistochemical detection of the p53 protein [5,6,11,12]. The gold standard elective surgery for UC with colitic cancer or high-grade dysplasia is proctocolectomy with ileal pouch anastomosis because synchronous and metachronous multiple cancers develop at high rates. However, we performed partial resection of the colon cancer in this UC case because the unaffected UC mucosa did not develop p53 mutations, nor was it likely to develop dysplasia or colitic cancer. It was an important issue that his UC had been well controlled with medicine. Among patients with moderate or worse inflammation of colitis that requires aggressive medical treatment, total proctocolectomy should be performed even in patients with sporadic cancer [3]. Also, he had 3 months from the initial diagnosis of UC to the operation of the cancer without previous history of UC. It could be a too short duration of colitis to develop colitic cancer. Recent review reported that the risk for developing CRC increased with longer duration of colitis, and CRC was rarely encountered in patients who have had colitis for less than 7 years [10].

Additionally, the differences in endoscopic, clinical, and histological findings between colitic and sporadic cancer have been investigated in previous studies, leading to the diagnosis of sporadic cancer [2,4]. The optimal procedure for UC with sporadic cancer remains unknown. Recent reports have shown the safety and efficiency of polypectomy treatment for adenoma-like dysplastic lesions, sporadic adenomas, and flat neoplasias in UC [14-16]. Uchino *et al*. have described that partial resection may be adequate compared with restorative proctocolectomy in view of anal function and quality of life [3]. We also support that partial resection is adequate for sporadic cancer in UC with well-controlled UC and shorter disease duration status.

GCAP was performed at 7 days after operation after his general condition had improved. Leukocytapheresis (LCAP) and GCAP therapies involve extracorporeal removal of peripheral leukocytes with the aim of suppressing harmful immunologic reactions in patients with chronic inflammatory disease. In adults with active UC, significant clinical improvement with LCAP and GCAP has been reported, permitting reduction of the doses of corticosteroids [17]. Ikeuchi *et al*. and Itabashi *et al*. have reported the effectiveness and safety of perioperative use of leukocytapheresis for postoperative complications [18,19].

## Conclusions

We have reported, for the first time, that a partial resection was performed in a patient with sporadic cancer with UC based on not only endoscopic, clinical, and histological findings but also that the unaffected mucosa did not detect p53 protein overexpression. In making the decision regarding a surgical procedure in colorectal cancer for patients with UC, we have to make a comprehensive evaluation based on the clinical, endoscopic, and histological findings which include immunostaining results. Although false positive and negative results in the analysis of p53 mutation status by immunostaining could be observed, we indicate that the immunostaining of p53 protein can be a useful marker.

## Consent

Written informed consent was obtained from the patient for publication of this case report and any accompanying images. A copy of the written consent is available for review by the Editor-in-Chief of this journal.

## Abbreviations

CRC: colorectal cancer
GCAP: granulocytapheresis
LCAP: leukocytapheresis
UC: ulcerative colitis
